# The Impact of Social Workers in Cirrhosis Care: a Systematic Review

**DOI:** 10.1007/s11938-022-00381-2

**Published:** 2022-04-19

**Authors:** Nneka N. Ufere, Jan Hinson, Simon Finnigan, Elizabeth E. Powell, John Donlan, Cathy Martin, Phil Clark, Patricia C. Valery

**Affiliations:** 1 Massachusetts General Hospital, 55 Fruit Street, Boston, MA 02114 USA; 2 Faculty of Health Sciences, School of Allied Health, Social Work Discipline, Australian Catholic University, Brisbane Campus (McAuley), 1100 Nudgee Road, Banyo, QLD 4014 Australia; 3 Centre for Functioning and Health Research, Metro South Health, Level 3, Buranda Village, Buranda, QLD 4102 Australia; 4 Department of Social Work, Princess Alexandra Hospital, 199 Ipswish Road, Woolloongabba, QLD 4102 Australia; 5 UQ Centre for Clinical Research, The University of Queensland, Herston, QLD 4029 Australia; 6 Department of Gastroenterology and Hepatology, Princess Alexandra Hospital, 199 Ipswish Road, Woolloongabba, QLD 4102 Australia; 7 Harvard Medical School, 25 Shattuck St, Boston, MA 02115 USA; 8 Queensland Liver Transplant Service, Princess Alexandra Hospital, 199 Ipswish Road, Woolloongabba, QLD 4102 Australia; 9 Population Health Department, QIMR Berghofer Medical Research Institute, 300 Herston Road, Herston, QLD 4006 Australia

**Keywords:** Chronic liver disease, Support services, Intervention, Psychosocial challenges, Supportive care

## Abstract

**Purpose of review:**

To report social workers’ involvement in supporting patients with cirrhosis.

**Recent findings:**

Six intervention studies (three published in the past 3 years) highlighed the potential role of social worker-led interventions to improve the outcomes of patients with cirrhosis. In studies of patients with alcohol-related liver disease (*n* = 4), social workers conducted psychosocial assessments, screened for substance use disorder and psychological distress, coordinated referrals to addiction services, and provided relapse prevention therapy. In studies including transplant recipients or candidates (*n* = 2), social workers focused on psychosocial interventions. In two studies (*n* = 1 patient with alcohol-related liver disease; *n* = 1 transplant recipients), social workers provided practical support (e.g., housing, transportation). Most articles provided limited information about the intervention and the role of the social worker, making comparisons of the studies difficult.

**Summary:**

More high-quality evidence is needed to formally assess the impact of social workers in improving the outcomes of patients with cirrhosis.

**Supplementary Information:**

The online version contains supplementary material available at 10.1007/s11938-022-00381-2.

## Introduction

Patients with cirrhosis experience significant psychosocial challenges that affect their health-related quality of life. The rates of alcohol use disorder, social isolation, depression, and anxiety are rising among patients with cirrhosis [1–3]. One in three adults with cirrhosis experiences financial hardship from medical bills, contributing to food insecurity, medication non-adherence, and frequent unplanned healthcare utilization in this population [4•]. Qualitative studies of patients with cirrhosis and their caregivers have underscored their unmet psychosocial care needs in the domains of illness and prognostic understanding, care coordination, coping with uncertainty and stigmatization, and caregiver support [5•, 6–8]. Prior work has highlighted that the psychosocial care needs of patients with cirrhosis are generally under-addressed in routine clinical hepatology care [5•, 9•, 10••].

Social workers, as part of multidisciplinary clinical care teams, play a key role in addressing psychosocial aspects of care in chronic disease management to deliver person-centered care for patients and families. Their clinical expertise and skill in working at the socio-ecological interface where the individual, their social context, and the environment are inextricably linked provides opportunity for social workers to improve patients’ health and their health experiences [11]. These skills may include psychoeducation, care coordination, case management, financial and/or other system navigation, community and/or service linkages, patient/family advocacy, and emotional support and counselling [12]. Recent clinical trials for patients with advanced cancer, kidney disease, and heart failure as well as their caregivers demonstrated that social worker-led interventions led to improved health and related outcomes across a range of factors. These included quality of life, depression and anxiety symptoms, coping, financial hardship, prognostic understanding, informed decision-making, and advance care planning [13–20]. While more recent work has highlighted the potential benefit of multidisciplinary team-based care for patients with cirrhosis, the specific role of social workers in the support and management of patients with cirrhosis has been chronically underexplored [21].

In this study, we aimed to systematically and critically review articles reporting social workers’ involvement in providing support to adult patients with cirrhosis. More specifically, this review addressed the following questions:

(i) What type of interventions have been used by social workers to improve outcomes for adult patients with cirrhosis?
(ii) What study endpoints were used in these studies (e.g., quality of life, health service use, support service use, unmet supportive care needs)?
(iii) What tools were used measure the study endpoints?

## Materials and methods

### Protocol and registration

This review was registered with the Center for Reviews and Dissemination at the University of York (PROSPERO registration number 241939). The review followed the Preferred Reporting Items for Systematic Reviews and Meta-Analysis (PRISMA) guidelines [22].

### Eligibility criteria

The following criteria, based on the PICOCS process framework [23], were used for study selection:

- *Study population*: adult patients (aged 18 years or older) diagnosed with cirrhosis
- *Types of interventions*: we included studies that described interventions which involved social workers to support patients and improve patient outcomes. Studies that included a mixed sample of health workers were eligible if they included social workers. Social workers had to be directly involved in intervention delivery for a study to meet inclusion criteria.
- *Comparators*: usual patient support or treatment or no patient support were identified as comparator.
- *Types of outcomes*: all outcomes were included, e.g., quality of life, health service use, support service use, unmet needs.
- *Context or setting*: hospital- and/or community-based health settings that deliver adult healthcare.
- *Study design*: original studies of any design, except case reports, were considered. Controlled trial designs (randomized/non-randomized interventions), pre- and post-intervention studies, qualitative and mixed methods studies were eligible for inclusion. Publications that were not data-driven (e.g., reviews, discussion documents), conference proceedings, or without an abstract were excluded.

Articles published in English, Portuguese, Spanish, French, and German published prior to 15 February 2021 were eligible.

### Information sources

The literature search was conducted from inception to 10 August 2021 using six electronic bibliographic databases, namely, CINAHL, PsycINFO, PubMed, Web of Science, Embase, and Social Services Abstracts. The search was complemented by manually reviewing the reference lists of retrieved articles for other articles of potential relevance.

### Search strategy

A master list of search terms tailored to each electronic database was generated. Titles, abstracts, and key words were searched for possible combinations of relevant terms for “cirrhosis” and “social worker.” The search strategy used for Web of Science was as follows: TS = (“social work*” OR psychosocial worker OR psychosocial OR welfare work* OR welfare officer OR caseworker OR case worker OR social care worker OR social care professional OR support care worker OR support worker OR case manage* OR social service staff OR social support OR social services professional* OR social care staff OR social care provider*) AND TS = ((liver AND cirrho*) OR end-stage liver disease). Initially, no limits were imposed on language.

### Data extraction and data analysis

All the identified citations were imported into EndNote X5.0 for data management. The titles and abstracts were reviewed manually. Articles were categorized as “not relevant” or “potentially eligible” according to the eligibility criteria. Articles considered “not relevant” were excluded. The full-text of all “potentially eligible” articles was retrieved for further screening.

The literature search was conducted by a medical librarian. Data management was conducted by one researcher (PCV). Nine researchers (CM, EEP, EO, JD, JH, NNU, PCV, PC, and SF) independently screened the titles and abstracts of publications against the eligibility criteria and selected “potentially eligible” publications for review (at least two reviewers per title). Any discrepancies in selecting articles were resolved by discussion among the researchers and consulting two other clinical staff involved in the study (EEP and NNU).

We used a structured data abstraction form to extract key information from each of the six articles and created tables to display and categorize the data. Data collated using a Microsoft Excel spreadsheet (Microsoft Corp, Redmond, WA, USA) included: author, year published and country, study aims, study design, number of patients included, type of health professionals involved in the intervention, study endpoints and measurement tools, the role of social workers in supporting patients (type of intervention), and major findings. A qualitative descriptive approach was utilized to review and synthesize the findings.

The methodological quality of eligible articles was assessed using a checklist created by Hawker et al. [24]. Each item of this checklist has a maximum score of 4 with a score of 1 indicating very poor and a score of 4 indicating good. Total maximum score is 36. We calculated total score and average scores.

## Results

### Systematic search

**Fig. 1:**
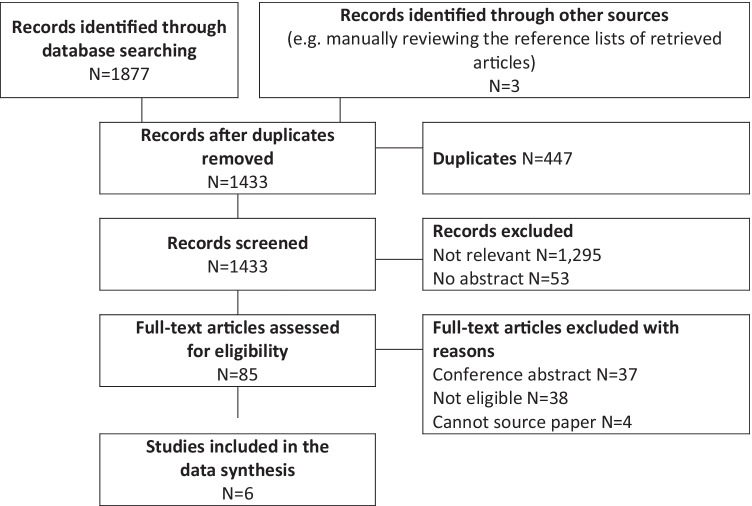
Flow diagram of search and selection of articles for review

The search of these six databases yielded 1877 citations in total (Fig. 1). After deleting 447 duplications, 1433 citations remained in the EndNote database for further screening. Three extra titles were identified by manually reviewing the reference lists of retrieved articles. One thousand four hundred thirty-three titles and abstracts were reviewed manually by two independent reviewers. One thousand two hundred ninety-five articles were categorized as “not relevant” according to the eligibility criteria by both reviewers, 85 were considered “potentially eligible” by at least one reviewer, and 53 did not have an abstract. Articles considered “not relevant” and with no abstract were excluded. The full-text of 81 “potentially eligible” articles was retrieved for further screening, and 4 titles could not be sourced. Of the 81 titles reviewed, 37 were conference abstracts and 38 were not eligible and therefore excluded. Six titles were included in the review, and three were published in the past 3 years.

### Characteristics of the reviewed studies

As shown in Table 1, most of the studies (4/6) in this review focused on interventions for alcohol disorder. Three were conducted in the USA, one in Denmark, and two in Canada. Studies included in the review were published between 1990 and 2021—two studies were conducted over two decades ago [25, 26], and three were published in the past 3 years [27••, 28, 29••]. Sample sizes ranged from 33 to 286. Three studies compared an intervention administered to a group of patients with usual care experienced by a control group of patients. In three studies, all patients who received the intervention were followed up with no control group (in one study, the data was collected retrospectively).

**Table 1:** Summary of studies included in the review

	Author, year published, and country					
	Kuchipudi et al. [25], 1990, USA	Zilberfein et al. [26], 2001, USA	Andersen et al. [34], 2013, Denmark	Verma et al. [29••], 2019, USA	Craig et al. [28], 2020, Canada	Carrique et al. [27••], 2021, Canada
Study aims	To assess the effect of a motivational intervention emphasizing the need for and the benefit of treatment for alcohol use disorder	To examine social work practices and psychosocial interventions with pre- and post-liver transplant patients and their families	To establish an outpatient rehabilitation clinic for patients with alcohol-related liver disease with a recent hospital admission for hepatic encephalopathy	To implement a behavioral health program in an ambulatory hepatology setting for alcohol use disorder, substance abuse, and depression, assess the acceptability to patients, and explore the effectiveness of this program in improving quality of life and reducing the targeted illnesses over time	To evaluate the use of a coping skills group therapy intervention conducted by transplant social workers qualified to provide psychosocial interventions aimed at decreasing depression and anxiety, and increasing healthy coping skills in a population of transplant candidates	To determine patient suitability for transplantation and risk for relapse through the use of selective criteria, to operationalize a multidisciplinary team of clinicians to assess and mitigate this risk, and to monitor for alcohol use both pre- and post-liver transplant and intervene when appropriate
Design	Interventional study with a control arm	A practice-based, retrospective observational study without a control group	A prospective study with a historical control group	A pragmatic quality improvement study to implement screening for alcohol use disorder, substance use disorder, or depression and referral to a behavioral intervention program in an ambulatory hepatology setting. No control group	Intervention study (pre-post design) comparing coping skills, depression, and anxiety symptoms pre- and post-intervention, and at 1 month follow-up. No control group	Pilot interventional study with a historical control arm
Number of patients included	114 patients admitted to hospital with cirrhosis (*n* = 71), ulcer, or pancreatitis who were currently drinking and not currently active in alcoholism treatment. Patients were randomly assigned to motivational interventional therapy group (*n* = 59) or control group (*n* = 55)	286 liver transplant recipients (29% with substance use problems pre-transplant) who had a psychosocial assessment done by social workers before and after transplant	19 patients with alcohol-related liver disease recently hospitalized with hepatic encephalopathy in outpatient rehabilitation clinic group and 14 historical controls with hepatic encephalopathy discharged 1 year prior to the intervention	95 patients with chronic liver disease who screened positive. Data was collected prospectively	Convenience sample of 16 patients awaiting kidney transplant and 25 awaiting liver transplant	44 patients with alcohol-related liver disease receiving the intervention and a liver transplant, and 111 historical controls (patients with alcohol-related liver disease receiving transplants with > 6 months of abstinence within our program in the 18 months before the institution of our pilot program)
Recruitment period	Not reported	1992–1994	2008–2010	2015–2016	2011–2013	2018–2020

Type of health professionals involved in the intervention	A lead clinician, a trainee clinician, a principal nurse on the floor or the gastroenterology fellow, and a social worker. Patients were also offered to participate in a group discussion with a psychiatric nurse therapist	Mostly social workers; over two-thirds of the patients also had a consultation with a psychiatrist	Nurse, a physician (if needed), and two employees from the Social Services of Copenhagen	Social worker trained in behavioral therapies. Some patients with severe symptoms received referrals to a behavioral health speciality (for alcohol and substance use disorder) or psychiatry (for depression) in addition to receiving the brief intervention	Social workers who were Master of Social Work (MSW)-level practitioners authorized to provide psychosocial interventions	A multidisciplinary team including hepatologists, addiction and consultation-liaison psychiatrists, social workers, and a nurse practitioner
Study endpoints	Outcome measures were utilization of alcoholism programs (inpatient or outpatient); attendance at three outpatient follow-up clinics in internal medicine or gastroenterology; and self-reported sobriety at about 10 weeks (interview on drinking status with the patient and with household companions)	Therapeutic social work and psychiatric interventions as well as concrete services were examined to determine frequency of use both pre- and post-transplant. Number and percentage of patients using services were described	The primary endpoints were 1-year survival and hospital readmissions. Economic costs of subsequent hospital admissions were a secondary outcome. Clinical, demographic and biomedical parameters, including severity of liver disease, and alcohol consumption	Primary outcomes were patient acceptability and change in quality of life from baseline to 3 months. Secondary outcomes included change in illness severity scores over time and sustainability of change in quality of life at 6 months	Study endpoints included coping skills; depression; and anxiety	Study outcomes included post-transplant survival and return to alcohol use
Measurement tools	The Comprehensive Drinker Profile questionnaire was used at baseline. No specific tool was used to assess sobriety at follow-up	No specific tools were used to measure study endpoints	No specific tool was used to measure alcohol consumption	The Chronic Liver Disease Questionnaire (CLDQ) [27••] was used to assess quality of life. Illness severity was assessed using validated questionnaires for alcohol use disorder (Alcohol Use Disorders Identification Test [AUDIT]) [28], substance use disorder (Drug Abuse Screen Test [DAST-10]) [29••], or depression (Patient Health Questionnaire-9 [PHQ-9]) [30]	The Brief COPE questionnaire with 14 coping-related subscales [35], Hamilton Depression Rating Scale [HAM-D] [36], and Hamilton Anxiety Rating Scale [HAM-A] [37] were used to assess study endpoints	Alcohol use post-transplant was assessed through patient self-report and biomarker testing
The role of social workers in supporting patients (type of intervention)	The motivational intervention included at least 3 separate discussions of the relationship of patient’s disease to continued drinking, namely: the patient’s health and drinking history was reviewed by the lead of the unit; 1 h later the trainee clinician reinforce the message; 2 days later the principal nurse or the gastroenterology fellow reviewed the patient and the need for therapy and support; a social worker discussed the available programs and facilities that may benefit the patient*; and lastly a group session with a psychiatric nurse *This component was needed for the intervention to be considered delivered	The specific interventions used by health workers were not described. All patients were assessed by all members of a multidisciplinary team (including a social worker) to determine their eligibility for liver transplant. The common reasons for social work consultation post-op included insurance problems, mental and behavioral symptoms, family problem, adjustment to new medical diagnosis, physical discomfort, and transportation	The specific interventions used by nurse, physician, and employees from the Social Services of Copenhagen were not described in the paper. Patients referred to the rehabilitation program were seen by a trained nurse within 1–3 weeks after discharge and by a physician if needed to assess patients’ clinical, psychological, and social problems including alcohol consumption. Two employees from the Social Services of Copenhagen collaborated closely with the rehabilitation clinic and addressed issues such as housing, economic, medical, and other needs for patients	Social workers provided a brief intervention (15–20 min) based on principles of motivational interviewing and cognitive behavioral therapy and targeted six elements: feedback on behavior and consequences, responsibility to change, advice, menu of options to bring about change, empathy, and self-efficacy for change. The social worker identified ambivalence, taught motivation and self-efficacy techniques, and coached the patient to build a commitment to change. For depression, the social worker offered cognitive behavioral therapy and facilitated conversations from negative to positive thoughts leading to changes in attitudes and behaviors. A repeat brief intervention occurred at 3 months	Qualified social workers provide psychosocial interventions aimed at enhancing patients’ repertoire of coping skills to allow them to better manage the psychosocial demands associated with the pre-transplant experience. The program was designed around cognitive-behavioral, narrative, and mindfulness interventions	The social worker met with each patient and their support people to assess the availability of the emotional and instrumental support typically needed for transplantation and conducive to alcohol abstinence. The specific tools used during this assessment were not described. The intervention (relapse prevention therapy), developed by addiction psychiatrists and an addiction therapist (a registered social worker with specific prior training in addiction therapy), consisted of 6 core sessions provided to all patients, and an additional 4 optional sessions. Booster sessions were available for patients who requested them and for those with severe alcohol-related liver disease
Major findings	Self-reported sobriety at ~ 10 weeks did not differ between the intervention and control group (37.8% vs. 37.5%, respectively). Attendance of two-to-three outpatient follow-up clinics was associated with sobriety (*p* < 0.01) The sample size was sufficient to provide 41% power to detect a 25% improvement in sobriety rate, and 82% power to detect a 50% improvement	There was an increase in the use of therapeutic social work and psychiatric interventions and concrete services from pre- to post-transplant. Of the interventions noted, the most frequently used post-transplant included: individual counselling (70%), family counselling (3.5%), liver transplant support group (32.9%), evaluated by a psychiatrist (32.2%). Of the concrete services noted, the most utilized post-transplant were assistance with insurance (15.4%), transportation (15.4%), and home care (36.7%)	1-year survival was 84% in the intervention group vs. 36% in the historical controls (*p* = 0.012). There was no difference in hospital readmissions and hospital costs between individuals in the intervention and historical control groups. The majority (17/19) of patients in the control group had decreased alcohol consumption	For the 95 patients who underwent the brief intervention, quality of life improved from baseline to 3 and 6 months (*p* < 0.001). AUDIT and DAST-10 scores also improved significantly at 6-month follow-up (*p* = 0.0048 and *p* = 0.038, respectively). Patients with depression had an improvement in their PHQ-9 scores by 3.7 points at 6 months (*p* < 0.0001) and their quality of life improved the most among the study participants. There was a significant improvement in the percentage of patients with severe disease scores on the AUDIT, DAST-10, and PHQ-9 scales between baseline and 6 months (*p* < 0.05). Depression was the only independent predictor of change in quality of life over time. Of the enrolled patients, 82% agreed the brief intervention improved their overall care and 87% indicated a desire to continue with the behavioral program	Over the 3 time periods, the pilot study showed that patients experienced increases in acceptance (*p* < 0.047) and religious coping strategies (*p* < 0.042), and decreases in denial (*p* < 0.039) and self-blame (*p* < 0.025). There was a reduction in anxiety and depression between pre- and post-intervention and between pre-intervention to 1 month follow-up (*p* values for these comparisons were < 0.001)	This pilot study showed no significant differences in survival rates (*p* = 0.07) and proportion of patients returning to alcohol use (6.8% vs. 16%, *p* = 0.21) between patients receiving transplants through the pilot program versus a historical control group with > 6 months of abstinence prior to transplant

### Type of interventions used by social workers to improve patient outcomes

#### Alcohol-related liver disease

Two studies implemented a motivational intervention (Table 1). In 1990, Kuchipidi et al. [25] implemented a motivational intervention for 114 hospitalized patients with untreated alcohol use disorder. Patients presenting to the hospital with a recurrent admission for alcohol-related liver disease (*n* = 71), peptic ulcer disease with gastritis, or pancreatitis were randomly assigned to a motivational intervention or control group. Patients assigned to the motivational intervention group participated in three sessions about the relationship between alcohol consumption and their health, including individually meeting with a social worker who discussed available relapse prevention programs that may benefit each patient. There were no significant differences in rates of alcohol abstinence between patients in the intervention and control groups at 10-week follow-up.

In 2019, Verma et al. [29••] implemented a brief motivational intervention for 95 outpatients with chronic liver disease who screened positive for alcohol use disorder, substance use disorder, and/or depression while awaiting their hepatology clinic appointments. Patients who screened positive were offered a brief motivational intervention delivered by trained social workers at the point of care and at 3 months. The social worker also coordinated referrals to behavioral health specialists (for alcohol and substance use disorder) and psychiatry (for depression) for patients with severe symptoms. Primary and secondary outcomes included change in health-related quality of life assessed by Chronic Liver Disease Questionnaire (CLDQ) [30] and changes in illness severity scores assessed using the Alcohol Use Disorders Identification Test (AUDIT) [31], Drug Abuse Screen Test (DAST-10) [32], and/or Patient Health Questionnaire (PHQ-9) [33]. For the patients who received the intervention, CLDQ scores improved significantly from baseline to 3 and 6 months (*p* < 0.001). AUDIT and DAST-10 scores also improved significantly at 6-month follow-up (*p* = 0.0048 and *p* = 0.038, respectively). Patients with depression had an improvement in their PHQ-9 scores by 3.7 points at 6 months (*p* < 0.0001) and significantly better improvement in quality of life.

In 2020, Carrique et al. [27••] reported the effects of a prospective pilot program involving integrated addiction treatment for 44 patients with alcohol-related liver disease (either severe alcoholic hepatitis or chronic alcohol-related liver disease) and less than 6 months of abstinence prior to undergoing liver transplantation. The study involved a specialized, multidisciplinary and colocalized team consisting of transplant hepatologists, addiction psychiatrists, a nurse practitioner, and social workers. The social workers conducted psychosocial assessments of 379 patients referred to the pilot program to assess their available emotional and instrumental supports and determine their suitability for the pilot program. Patients accepted to the pilot program and meeting criteria for alcohol use disorder were required to participate in a relapse prevention therapy program developed by the team’s addiction psychiatrists and an addiction therapist (a registered social worker with specific prior experience and training in addiction therapy). This program consisted of 6–10 individual sessions (in-person, over the phone, and/or through other virtual means) covering the core components of relapse prevention therapy. The addiction therapist also pre-emptively assessed treatments that patients accessed at outside centers to ensure that they were evidence-based and appropriate. In total, 44 patients in the pilot program were transplanted over the study period. There were no significant differences in survival rates for the patients transplanted through the pilot program compared to a historical control group of 111 patients with more than 6 months of abstinence prior to receiving a transplant. Only 3 (6.8%) patients in the pilot program returned to alcohol use after transplant within an average of 260 days post-transplant compared to a rate of relapse of 16% for patients in the historical control group (*p* = 0.21).

In 2013, Andersen et al. [34] reported the effects of an outpatient rehabilitation clinic for 19 patients with alcohol-related liver disease who had a recent hospital admission with hepatic encephalopathy. Patients were seen by a nurse, a physician as needed, and employees from the Social Services of Copenhagen who were involved in the study and took a special interest in the patients. The specific interventions used by social workers were not reported (see Table 1 for brief summary of psychosocial domains addressed). One-year survival was significantly higher in the intervention group compared to a historical control group of 14 patients who had a hospital admission for hepatic encephalopathy 1 year prior to the intervention. The study authors posited that the special attention from the Social Services staff regarding issues related to housing and economic conditions may have indirectly contributed to the positive outcomes in the intervention group.

#### Liver transplant candidates

Two studies of patients who received liver transplant or were transplant candidates focused on psychosocial interventions. In 2001, Zilberfein et al. [26] conducted a retrospective study based on medical chart review of 286 liver transplant recipients who had a psychosocial assessment done by social workers before and after transplant. In this study, they showed a substantial increase in the use of therapeutic social work and psychiatric interventions and social services in the post-transplant setting (compared to pre-transplant), with an increase in the use of individual counselling (70% vs. 42%), family counselling (53% vs. 33%), assistance with transportation (15% vs.4%), and assistance with home care (37% vs. < 1%). The specific interventions used by social workers were not reported and patient outcomes were not assessed.

In 2020, Craig et al. [28] piloted a coping skills group intervention for patients awaiting kidney (*n* = 16) or liver (*n* = 25) transplantation at a single transplant program. Two transplant social workers led an 8-week psychoeducational group intervention to enhance patients’ coping skills to allow them to better manage the psychosocial demands of the pre-transplant experience. The study used a pre-post design to assess coping skills (Brief COPE) [35], depression symptoms (Hamilton Depression Rating Scale [HAM-D]) [36], and anxiety symptoms (Hamilton Anxiety Rating Scale [HAM-A]) [37] pre-intervention, post-intervention, and at 1-month follow-up. On pre-post testing, the patients had significantly decreased use of dysfunctional coping (self-blame and denial) and increased use of emotion-focused coping (accepting the reality of the situation, finding comfort in religious/spiritual beliefs) and problem-focused coping (getting help or advice from other people). Anxiety and depression scores were significantly reduced and these changes were sustained at 1-month follow-up.

### Study endpoints and measurement tools used

Study endpoints varied across the reviewed studies. Three studies focused on health service utilization, solely [26] or in combination with clinical or behavioral outcomes (e.g., survival [34], self-reported alcohol use [25]). One study examined change in quality of life and illness severity score [29••]. One study examined clinical or behavioral outcomes (survival and relapse of alcohol use) [27••]. The Chronic Liver Disease Questionnaire (CLDQ) [30] was the only validated disease-specific tool used to measure a study endpoint for patients with liver disease—this was utilized in Verma et al.’s study. Craig et al.’s study used three generic tools to assess the impact of the intervention, namely, the Brief COPE questionnaire [35], the HAM-D [36], and the HAM-A [37].

### Quality of studies

Nine domains were assessed from the six studies included in this review, namely, abstract and title; introduction and aims; method and data; sampling; data analysis; ethics and bias; findings/results; transferability/generalizability; and implications and usefulness. All studies were assessed by at least two researchers. Using Hawker et al. [24] quality assessment tool, studies were assessed as either good, fair, poor, or very poor in the reporting of details for all abovementioned categories. We did not exclude studies based on a cutoff score on this checklist. Details about the assessments are shown in Supplementary Table 1. Average scores ranged from 2.7 (fair-poor) [34] to 3.5 (good-fair) [25]. Three studies were rated as very poor and/or poor for ethics and bias [26, 29••]. The study with the lowest score [34] was rated fair-poor or poor for 5 categories (method and data, sampling, data analysis, ethics and bias, and transferability/generalizability).

## Discussion

Patients with cirrhosis and their caregivers have substantial unmet psychosocial care needs. It is in this context that we conducted the first systematic review of the current literature related to the role of social workers in addressing the psychosocial needs of adult patients with cirrhosis. Our review demonstrated that there is a paucity of published data on the impacts of social workers to improve the outcomes of patients with cirrhosis. An extensive and methodical review of 1433 articles identified from six databases resulted in the identification of only six relevant studies.

Despite the limited number of studies included in the review, the studies highlighted the potential role of social worker-led interventions to improve the outcomes of patients with cirrhosis. In studies of patients with alcohol-related liver disease, social workers conducted psychosocial assessments, screened for substance use disorder and coincident psychological distress, coordinated referrals to addiction services, and provided relapse prevention therapy [25, 27••, 29••, 34]. In two studies, social workers provided instrumental support for patients with cirrhosis through providing housing, transportation, and financial assistance [26, 34].

In four studies, social workers delivered behavioral interventions that included coping skills group therapy, cognitive behavioral therapy, brief motivational interventions, and patient/family counselling to address the psychological needs of patients with cirrhosis and their caregivers [26, 27••, 28, 29••]. In the included studies, social worker-led interventions integrated into routine hepatology care may have contributed to an improvement in health-related quality of life, alcohol and substance use, depression severity, and coping for patients with cirrhosis.

The impacts on health outcomes of social worker-led interventions have been evaluated in other chronic disease populations. Two clinical trials within oncology demonstrated that the use of social workers as financial navigators to alleviate the burden upon patients and caregivers of medical and non-medical costs resulted in their improved access to financial assistance with housing, utilities, and transportation [19, 20]. Randomized controlled trials assessing the role of social workers in delivering psychosocial interventions to caregivers of patients with cancer have demonstrated efficacy in improving caregiver burden, quality of life, depression, anxiety, self-efficacy, and coping [15, 16]. In nephrology, social worker-led group education interventions have improved knowledge about renal replacement therapy and live donor kidney transplantation and informed decision-making among patients with chronic kidney disease and their families in two randomized controlled trials [17, 18]. Lastly, in cardiology, two randomized controlled trials involving social workers delivering symptom management and palliative care interventions led to improvements in depressive symptoms, fatigue, prognostic understanding, and advance care planning documentation among patients with heart failure [13, 14].

**Fig. 2:**
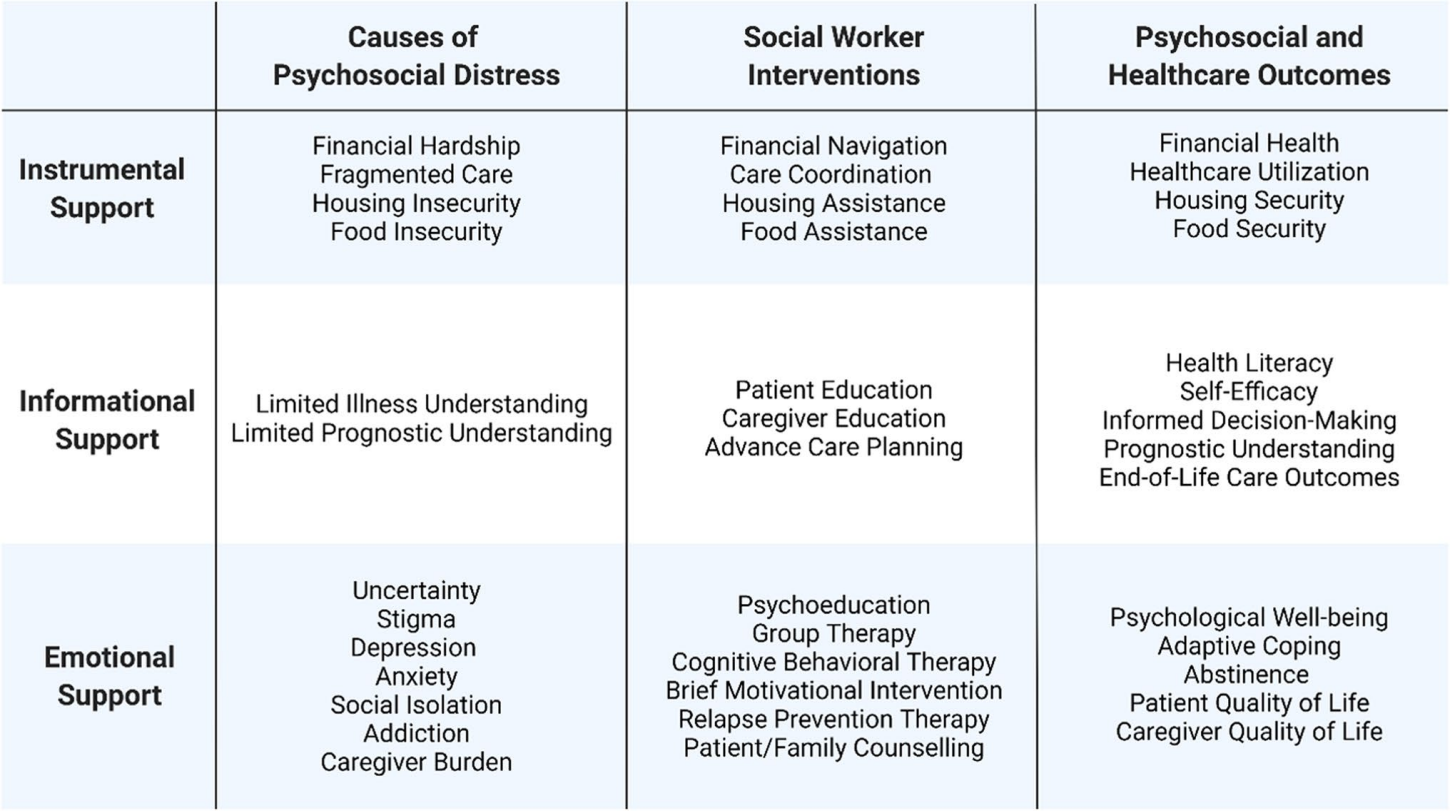
Categories of psychosocial care needs of patients with cirrhosis and examples of social worker interventions

More high-quality research is needed to examine the potential impact of social worker-led interventions on the psychosocial and health outcomes of patients with cirrhosis and their caregivers. The limited existing literature predominantly focuses on the role of social workers in supporting the needs of patients with alcohol-related liver disease. Patients with non-alcoholic fatty liver disease, who have high rates of psychological distress and food insecurity, may be a population that could particularly benefit from psychosocial support delivered by social workers [38, 39]. Examining the role of social workers in addressing the financial, logistical, sociolegal, and emotional challenges that patients with cirrhosis and their caregivers face remains an area that requires further investigation. Future research should incorporate the use of psychosocial assessment instruments; the Supportive Needs Assessment tool for Cirrhosis is a validated instrument that assesses the psychosocial care needs of patients with cirrhosis and has been found to be highly correlated with health-related quality of life in this population [10••]. Figure 2 illustrates the categories of psychosocial care needs of patients with cirrhosis and examples of social worker interventions. Outcomes of future studies involving social worker-assisted interventions should include healthcare utilization, quality of life, financial health, health literacy, self-efficacy, informed decision-making, prognostic understanding, engagement in advance care planning, caregiver outcomes, and/or psychological well-being.

### Strengths and limitations

A search of six relevant electronic bibliographic databases gives this review breadth and comprehensiveness. A minimum of two researchers assessed the titles and content of publications against the eligibility criteria and the quality of eligible articles. While the ability to assess studies written in four languages other than English decreased potential selection bias, there is the possibility that some relevant studies may have been missed. A key limitation is that most publications provided limited information about the intervention and often lacked details regarding the role of the social worker making comparisons of the studies difficult. Other limitations to this systematic review that need to be acknowledged include small sample sizes; narrow focus (e.g., alcohol misuse); lack of consistent measurement of outcomes; and two studies scored low (fair or poor) on their quality assessment score. Only one study, by Kuchipudi et al., utilized a randomized control trial. Due to the heterogenous outcomes and limited quantitative analyses in the included studies, we were unable to perform any meta-analysis.

Additionally, by only including studies that involved the use of social worker-led interventions, we may have missed other potential roles social workers can play in improving patient outcomes. For example, Mellinger et al. reported the successful implementation of an intervention led by a multidisciplinary team for patients with alcohol-related liver disease [40••]. While this study was not eligible for inclusion in this review, the multidisciplinary team included a social worker who was involved with making a pre-treatment clinic phone call to prospective participants largely to alleviate barriers of attendance to the clinic program. In this study, the psychologist and psychiatrist provided the one-on-one sessions and referred patients to groups or to inpatient/outpatient rehabilitation. The social worker played a key role in proactively addressing barriers to potential patient recruitment into the clinic, which could be considered a part of intervention delivery. The ability to reduce barriers to healthcare access for patients with cirrhosis is an important potential role of social workers that should be formally examined in future work.

## Conclusions

Despite a paucity of data, this systematic review highlighted a promising role for social workers in addressing the psychosocial aspects of care of patients with cirrhosis. More high-quality evidence is needed to assess the impact of clinical social workers as a part of an integrated hepatology care team in improving the health outcome of patients with cirrhosis.

## Supplementary Information

Below is the link to the electronic supplementary material.

Supplementary file1 (DOCX 18.9 KB)

## Abbreviations

AUDIT: Alcohol Use Disorders Identification Test
CLDQ: Chronic Liver Disease Questionnaire
DAST-10: Drug Abuse Screen Test
HAM-A: Hamilton Anxiety Rating Scale
HAM-D: Hamilton Depression Rating Scale
PHQ-9: Patient Health Questionnaire
PRISMA: Preferred Reporting Items for Systematic Reviews and Meta-Analysis guidelines
