# Supplementary material for: The Impact of Social Workers in Cirrhosis Care: a Systematic Review
Source: Curr Treat Options Gastroenterol. Author manuscript; Available in PMC 2022 Jun 6. (PMC9167183; doi:10.1007/s11938-022-00381-2)
Supplement: Supplementary Material [file NIHMS1801355-supplement-Supplementary_Material.docx]

**Supplementary Table 1**. Assessment of study quality according to Hawker et al. scoring

| **Author, year published** | **Kuchipudi et al., ^(25)^ 1990** | **Zilberfein et al, ^(26)^  2001** | **Andersen et al., ^(34)^  2013** | **Verma et al., ^(29)^**  **2019** | **Craig et al., ^(28)^**  **2020** | **Carrique et al., ^(27)^**  **2021** |
| --- | --- | --- | --- | --- | --- | --- |
| **Abstract and title** | **Good 4/Good-Fair 3.5**  Clear title. Abstract was thorough. | **Fair 3/Fair-Poor 2.5**  Clear title. Abstract very short (may be due to journal guidelines) and with no background or mention of findings. | **Good 4/Fair 3**  Abstract has most of the information but could have given more details regarding social work/services intervention. It could have noted social work/services intervention in the title. | **Good 4/Good 4**  Clear title. Detailed information in Abstract. | **Good 4/Fair 3**  Clear title. Abstract included most of the information. Methods and results could have been described in more detail. | **Fair 3/Fair 3**  Clear title. Abstract could have described the intervention in more detail. |
| **Introduction and aims** | **Fair 3/Fair 3**  Limited background, clear hypothesis and study objectives however limited discussion of intervention itself. Could have described both the hypothesis and aims more clearly. | **Good 4/Good 4**  Comprehensive background with clear highlights of gaps in literature. Aims and objectives stated in beginning of Methodology section. | **Fair 3/Fair 3**  Very brief, although possibly due to word limit. It doesn't mention social work/services. | **Good 4/Good 4**  Enough detail but succinct. Aims and objective stated. | **Good-Fair 3.5/Good-Fair 3.5**  Detailed background and rationale for present study. Aims were not stated in a clear, concise way. Hypothesis clearly stated. | **Good 4/Good 4**  Aims and objectives clearly stated. Good background and reason for conducting the study was well articulated. |
| **Method and data** | **Fair 3/Fair 3**  Included information about the tool used to measure alcohol use at baseline. Limited description of the methods used for outcome evaluation (e.g. unclear how alcohol use was measured at follow up). Lacked detailed description of the intervention, blinding, and reasons for limiting follow up to 5 months. | **Good-Fair 3.5/Fair 3**  Inclusion criteria, measurements, data source are well described. No mention of data analysis in relation to results. Details re-“psychosocial information retrieval form” and “Reliability testing” were lacking. | **Fair 3/Fair-Poor 2.5**  No mention of how alcohol consumption was measured; no description of the social work/ services intervention; was social workers involved? Could have given examples re economic- or housing-related supports offered. | **Good 4/Fair 3**  Clear and detailed descriptions. Screening questions available in ‘Supporting Material Appendix 1’. Scales e.g. PHQ-9 not included but this is a standard tool. | **Good-Fair 3.5/Fair 3**  Inclusion criteria clearly described. Intervention, coping skills group therapy, were described, but description could be better. Mentions an extra questionnaire that only some participants received at the end of the group, but doesn’t explain what the questionnaire included or why not all patients received it. | **Good 4/Good 4**  Clear details about eligibility and exclusion criteria. Pre and post-transplant assessment clearly described. |
| **Sampling** | **Good-Fair 3.5/Fair 3**  Details of study participants, response rates and discussion of sample size was provided. All patients admitted were potentially eligible. Eligibility/exclusion criteria were described. The study setting (where, when) was not reported. | **Good 4/Good 4**  Well described. Retrospective review of patients who received a first liver transplant during the study period. | **Poor 2/Poor 2**  Unclear whether all eligible patients were recruited/invited to participate; poor description of demographic characteristics of participants; sample size not justified/discussed; response rate not reported. Pilot-like nature of study noted. | **Good 4/Fair 3**  Addresses all criteria for ‘Good’ including details regarding methods, exclusions and response rates. No relevant information missing. | **Good 4/Fair 3**  A convenience sample of kidney and liver patients was used. Inclusion criteria clearly explained. Description of the recruitment process could be better. | **Good 4/Good 4**  Well described. Detailed description of case ascertainment, eligibility and exclusion criteria. |
| **Data analysis** | **Good 4/Good 4**  The data analysis was simple/very descriptive, but nevertheless appropriately reported. | **Fair 3/Fair 3**  Descriptive discussion of analysis scattered throughout findings. Very descriptive analysis (number and percentages, no p-values reported). | **Fair 3**/**Poor 2**  Data analysis not described in the Methods section. Brief descriptive of analysis scattered throughout findings. Other than log rank test mentioned in the survival plot, unclear what tests were used to compare groups. | **Good 4/Fair 3**  Sufficiently detailed. Discussion of when, and why, parametric vs non-parametric analysed performed. Significance levels noted. | **Fair-Poor 2.5/Fair-Poor 2.5**  No description of data analysis in methods, but scattered throughout results without much detail. | **Good 4/Good 4**  Sufficiently described in detail. |
| **Ethics and bias** | **Good 4/Good 4**  Ethics approval was obtained. No informed consent were obtained from study participants, but participants could refuse parts of the interview/assessments and intervention. | **Very poor 1/Very poor 1**  No discussion or mention of ethical approval, data protection/privacy, or consent. | **Fair 3**/**Poor 2**  Ethics approval was obtained. Informed consent was obtained from participants taking part in the intervention. No mention whether consent was obtained from controls. | **Poor 2/Very poor 1**  Study was stated to be quality improvement study (this noted in abstract and body of the manuscript), thus ethics approval not discussed. Confidentiality, potential bias and relationship between researchers and participants not discussed. Noted that 10 patients declined to participate. | **Fair 3/Fair 3**  Ethics approval obtained. No mention of patient consent except in abstract. No mention of privacy or data protection. | **Very poor 1/Very poor 1**  No mention of ethical approval, data protection/privacy, or consent. Patients were asked to sign an abstinence contract combined with consent to grant permission for random biomarker testing pre and post-transplantation. Unclear whether consent was obtained from historical controls. |
| **Findings/**  **results** | **Good 4/Good 4**  Tables clearly explained in the text. Results relate to aims and sufficient data is presented to support the findings. | **Fair 3/Fair-Poor 2.5**  Very descriptive. Tables well described in the text, but often included discussion and background that do not come directly from results. | **Fair 3/Fair 3**  Tables and figures are partially explained in the text. Results relate to aims. Health economic results were partially discussed in the text. | **Good 4/Good 4**  Details presented in text, tables and graphs. | **Fair 3/Fair-Poor 2.5**  Results are very descriptive. Bar graph is clear but lacks detail.  Results relate to aims. | **Good 4/Good 4**  Tables and figures are clearly explained in the text. Results relate to aims and sufficient data are presented to support the findings. |
| **Transferability/**  **generalizability** | **Good-Fair 3.5/Fair 3**  Provided context by describing other studies but limited description of sampling. | **Good 4/Good 4**  Context and setting allows comparability with other studies. | **Poor 2/Poor 2**  Small study. Limited generalizability and little information about the study setting. | **Fair 3/Fair 3**  Single-centre study. More details regarding clinic site (e.g. medical-only vs. multi-disciplinary) would be useful. Restricted generalisability noted. | **Fair 3/Poor 2**  Small single-center pilot study without a control group. Convenience sampling. Limitations explored in discussion section. Context and setting of the study is described sufficiently to allow comparisons with other settings. | **Poor 2/Poor 2**  Small single-centre pilot study. Restricted generalisability noted. |
| **Implications and usefulness** | **Good 4/Fair 3**  Highlighted differential loss to follow-up in this population, role of informed consent which could affect future research in this area, and had clear implications for practice. Contributed to knowledge, but not clear why intervention led to no difference between the two groups. | **Good 4/Good 4**  Direct implications and modifications made to their post-transplant screening protocol as a result of findings are discussed in “Conclusions” section. | **Fair 3/Fair 3**  Implications for policy/practice not discussed. The paper describes an insight/perspective about an intervention and suggests that further research is needed on this topic. | **Good 4/Good 4**  Addresses all three criteria for ‘Good’, namely contribution to new knowledge, discusses implications for practice and the need for further research. | **Fair 3/Good-Fair 3.5**  Implications are clearly discussed. Claims that findings support using psychoeducational intervention groups to help patients awaiting transplant cope with anxiety and depression. However, the study intervention was not clearly described and there was no control group. | **Fair 3/Fair 3**  The paper describes a pilot intervention and suggests that their assessment and intervention provide a framework for more equitable transplant care for all patients with alcohol-related liver disease. |
| **Total* and average score** | **31.8**  **Average score 3.5**  **(Good-Fair)** | **28.8**  **Average score 3.2**  **(Fair)** | **24.3**  **Average score 2.7**  **(Fair-Poor)** | **31.0**  **Average score 3.4**  **(Good-Fair)** | **31.8**  **Average score 3.5**  **(Good-Fair)** | **29.0**  **Average score 3.2**  **(Fair)** |

*Average scores for each item were added to estimate the total score
